# Comparing patient-reported outcomes across countries: An assessment of methodological challenges

**DOI:** 10.1177/1355819621990696

**Published:** 2021-02-07

**Authors:** Jason M Sutherland, Shanika Rajapakshe, Trafford Crump, Andrée Chartrand, Guiping Liu, Ahmer Karimuddin

**Affiliations:** 1 Professor, Centre for Health Services and Policy Research, School of Population and Public Health, University of British Columbia, Vancouver, Canada; 2 Research Assistant, School of Population and Public Health, University of British Columbia, Vancouver, Canada; 3 Assistant Professor, Department of Surgery, Cumming School of Medicine, University of Calgary, Calgary, Canada; 4 Research Assistant, Lawrence S Bloomberg Faculty of Nursing, University of Toronto, Canada; 5 Statistician, School of Population and Public Health, University of British Columbia, Vancouver, Canada; 6 Clinical Associate Professor, Section of Colorectal Surgery, Department of Surgery, University of British Columbia, Vancouver, Canada

**Keywords:** hernia, patient-reported outcomes, surgery

## Abstract

**Objectives:**

There is little published literature on the comparison of patient-reported outcomes between countries. This study aimed to assess pre- and postoperative health among samples of patients undergoing elective groin hernia repair procedures in the National Health Service (NHS), England, and groin hernia patients in Vancouver, Canada.

**Methods:**

We used datasets from two different sources. For the English NHS we used published anonymized patient-level data files which include the EQ-5D(3L) patient-reported outcome measure and a number of demographic and clinical characteristics. For Vancouver, we used data from a sample of Vancouver patients who completed the same instrument during a similar time frame. English patients were matched with Vancouver participant’s characteristics using propensity score methods. A linear regression model was used to measure differences in postoperative visual analogue scale values between countries, adjusting for patient characteristics.

**Results:**

Our study revealed a range of methodological issues concerning the comparability of patient-reported outcomes following hernia repair surgery in the two health systems. These related to differences in approaches to collecting patient-reported outcome measures and the nature of explanatory variables (self-report vs. administrative data), among other challenges. As a consequence, there were differences between the matched samples and the NHS data, indicating a healthy participant bias. Unadjusted results found that Vancouver patients (N = 280) reported more problems in domains of mobility, self care, usual activities and anxiety/depression than the matched cohort of NHS patients (N = 840). Interpreting differences is challenging given different sampling designs.

**Conclusions:**

There are significant hurdles facing comparisons of surgical patients’ outcomes between countries, including adjusting for patient differences, health system factors and approaches to survey administration. While between-country comparisons of surgical outcomes using patient-reported outcomes shows significant promise, much work on standardizing sampling design, variables and analytic methods is needed.

## Introduction

Patient-reported outcomes measures (PROMs) were originally designed to capture the perspective of participants of clinical trials on changes in symptoms, with the Food and Drug Administration in the United States defining PROMs as a report by a patient about a health condition, treatment benefit or risk in medical product trials.^1–3^ From these applications grew the measurement of general health and the expansion of PROMs into measuring population health,^4^,^5^ with a range of health status and preference-based PROM instruments developed since, such as the SF-36 (a health status instrument) and the EQ-5D-3L (a preference-based instrument).^6^,^7^

The concept of measuring health, or, more specifically, a change in health resulting from a health care intervention or quality improvement initiative, appealed to health policy makers, administrators, and providers in many settings.^8–11^ The motivation was to identify variations in PROMs, promoting those practices that resulted in the greatest net benefit to health, and eliminating those that did not, acting as a proxy for patients’ perceptions of effectiveness, and as a means to compare health care providers’ performance. The National Health Service (NHS) in England was a forerunner in collecting PROMs for this purpose,^10^ initiating, in 2009, the population-based collection of PROMs for four elective procedures: varicose veins, hip replacement, knee replacement, and groin hernia repair, asking patients aged 12 and over receiving treatments for these conditions to complete the EQ-5D(3L) pre- and postoperatively. The programme demonstrated that the wide-scale collection of PROMs was feasible.^8^,^12^

The use of PROMs as a performance measure to compare health care providers is not straightforward, however, and a range of methodological issues have been identified, including case-mix adjustment, acceptability among providers, and patients’ perceptions of their care.^13–17^ At the same time, there is considerable interest in using PROMs for benchmarking countries’ surgical outcomes internationally,^18^ and so help inform policy makers about the impact of health care reforms on patient outcomes^19^ and identify policies and delivery system characteristics of high performing countries.

There is, at present, no single administrative umbrella that would coordinate the standardization, reporting and use of PROMs between countries, and the feasibility of using PROMs for cross-country comparison is not well understood outside of multi-national clinical trials which operate under a uniform protocol,^20^,^21^ with the possible exception of work by Gordon et al.^22^ This study explores the feasibility of conducting cross-country comparisons of PROMs using data from the Vancouver Coastal Health Authority (VCH), a health care system located in British Columbia, Canada, which has been collecting PROMs from patients undergoing elective surgical hernia repair since 2013. The range of PROMs collected by VCH from these patients includes the EQ-5D(3L), an instrument which is also used in the English NHS to collect outcomes data from patients undergoing the same surgery. Specifically, we explore the methods necessary to undertake an international comparison of PROMs using prospectively collected, self-reported pre- and postoperative PROMs data from patients undergoing elective groin hernia repair procedures in the NHS and VCH.

## Methods

### NHS hernia data, England

In England, all providers of NHS-funded groin hernia surgeries are expected to encourage patients to complete the EQ-5D(3L) instrument pre- and postoperatively. The preoperative EQ-5D(3L) is administered between the time a patient is deemed fit for surgery and the procedure taking place, with local discretion determining the point in time when the instrument is administered.^23^ The postoperative EQ-5D(3L) is collected at least three months after the date of surgery.^23^ Data are publicly available as anonymized patient-level data files, containing: provider codes, sex, age group, procedure name, pre- and postoperative EQ-5D(3L) items, patient reported comorbidity information, and predicted changes in PROMs scores calculated from case-mix models.^23–25^

This study used NHS data from fiscal year 2015/2016, reporting 69,222 hernia repairs. Of these, 39,706 (57.4%) returned their preoperative EQ-5D(3L), of whom 24,812 (63.6%) returned their postoperative EQ-5D(3L). Response rates were consistent with statistics from previous years.^26^ Case-mix adjusted predicted scores derived from patients’ EQ-5D(3L) scores linked to corresponding hospital episodes from the NHS Hospital Episodes Statistics (HES) dataset. Patients’ whose hernia repairs and completed EQ-5D(3L) could not be linked to the HES data were not available for analyses; our analysis was thus based on a publicly available dataset of 20,059 hernia repairs with EQ-5D(3L).^27^

### Vancouver coastal health authority hernia data, Canada

The VCH dataset contains data on patients from 14 general surgeons in four Vancouver hospitals who have agreed to have their patients contacted to complete the EQ-5D(3L).^28^ Eligibility criteria include being 19 years of age or older, residing in the community, and ability to respond to survey questions in English, with or without assistance. Preoperative EQ-5D(3L) are administered at the time patients are placed on the surgical registry (the wait list for hernia repair surgery). Postoperative EQ-5D(3L) are administered six months after the date of surgery.

VCH provided an anonymized patient-level data file for analysis, which included demographic characteristics and EQ-5D(3L) items. To align VCH hernia patients as closely as possible to NHS groin hernia patients, we mapped operating procedure codes system version four (OPCS-4) codes to the Canadian Classification of Health Interventions (CCI) volume three codes, a Canadian-based taxonomy of interventions. We only included VCH patients with CCI codes that corresponded to the NHS OPCS-4 codes (see Online Supplement for further detail).

In this study we used VCH data from postoperative EQ-5D(3L) completed between October 2013 and October 2017. Of the 659 eligible hernia repairs during that period, 369 (56%) returned their preoperative EQ-5D(3L), of which 280 (76%) completed the postoperative survey. Participants’ EQ-5D(3L) were linked to hospital discharge summaries to ascertain participants’ sex, age and comorbidities.

For comparative analyses, VCH participants’ continuously valued age was categorized using the same categories reported in the published patient-level NHS data. The NHS dataset includes the following patient-reported comorbidities as indicator variables: heart disease, high blood pressure, stroke, circulatory problems, lung disease, diabetes, kidney disease, nervous system disease, liver disease, cancer, depression, and arthritis. In order to match VCH comorbidities to those present in the NHS data, the International Statistical Classification of Disease and Related Health Problems, Version 10, Canada (ICD-10-CA) corresponding to the above conditions were identified from patients’ hospital discharge summary (see Online Supplement for further detail on the matching procedure).

### Measurement instrument

Participants in both countries completed EuroQoL’s EQ-5D(3L) to measure general health as assessed through five items: mobility, self-care, usual activities, pain/discomfort, and anxiety/depression.^7^ Each item has three levels: no problem, moderate problems, and severe problems. The instrument also includes a vertical visual analogue scale (VAS), on which respondents rate their overall health on a continuous scale ranging from 0 (‘Worst imaginable health state’) to 100 (‘Best imaginable health state’). The EQ-5D(3L) was initially created with cross-national comparisons in mind and has been used in comparative analyses.^29^,^30^

### Analysis

The primary analyses sought to assess whether there were differences between the NHS and VCH in patients’ preoperative EQ-5D(3L) and/or postoperative EQ-5D(3L), and whether the gain in health attributable to hernia repair differed between countries. Participants under the age of 19 were not observed in the VCH data, and patients over the age of 89 years were not observed in the NHS data and we therefore excluded patients in these age ranges.

We applied a matched cohort design, matching VCH’s participants’ characteristics with a sample of the NHS HES patients. Matching probabilities were calculated using a logistic regression model to determine whether a patient was treated in VCH adjusting for sex, age category, and comorbidities. Each patient treated in VCH was matched to three NHS patients with the closest propensity of having been treated in VCH. Closeness was calculated using optimal nearest neighbour matching.

We analysed the pre- and postoperative EQ-5D(3L) by comparing responses to the five domains and the VAS, specifically, the proportional number of participants reporting no problems, moderate problems, and severe problems for each domain and at pre- and postoperative timepoints. The preoperative and postoperative mean VAS scores were summarized and compared using paired t-tests. Subgroup summaries were also created according to the age, sex, and comorbidity categories.

To evaluate whether the gain in health attributable to hernia repair differed between countries, a linear regression model of the EQ-5D(3L) VAS was used to adjust for available characteristics. After matching, no patients reported data for stroke, liver disease, depression, or arthritis; these comorbidities reported by the NHS were not included. An indicator variable was defined for whether a participant had their age and sex information withheld by the NHS (to ensure confidentiality) and another indicator variable for the patient’s health system (NHS or VCH). Goodness of fit was evaluated through visual inspection of residuals and the Akaike Information criterion (the AIC statistic). Additional missing data unrelated to the withheld age and sex information was found in both data sets, which was assumed to be missing at random and we therefore applied multiple imputation by chained equations.

### Ethics review

The study was approved by the University of British Columbia’s Behavioural Research Ethics Board.

## Results

Table 1 shows summary statistics for VCH participants and the matched sample of NHS patients. Matched samples were very similar on observed characteristics, while there were differences between the demographic characteristics and comorbidity profiles of unmatched NHS patients with VCH participants. This was expected given our optimal nearest neighbour matching approach. In VCH, there were no reported groin hernia repairs for participants older than 79 years of age whereas the NHS data recorded over 8% of groin repairs among patients in this age group. Observed sex differences were likely confounded by the masking of some patients’ sex and age in the NHS dataset.

**Table 1.** Summary statistics for VCH and NHS study populations.^a^

Sample characteristic	VCH	NHS (matched)	NHS (unmatched)
Overall	280 (100%)	840 (100%)	20,059 (100%)
Age category			
20–29	2 (0.7%)	3 (0.4%)	33 (0.2%)
30–39	5 (1.8%)	13 (1.5%)	243 (1.5%)
40–49	20 (7.1%)	53 (6.3%)	1314 (7.9%)
50–59	51 (18.2%)	123 (14.6%)	3078 (18.5%)
60–69	98 (35.0%)	323 (38.5%)	5703 (34.2%)
70–79	104 (37.1%)	325 (38.7%)	4949 (29.7%)
80–89	0 (0.0%)	0 (0.0%)	1345 (8.1%)
Withheld	0 (0.0%)	0 (0.0%)	3394
Sex			
Male	235 (83.9%)	747 (88.9%)	16,464 (98.8%)
Female	45 (16.1%)	93 (11.1%)	201 (1.2%)
Withheld	0 (0.0%)	0 (0.0%)	3394
Comorbidity			
Heart disease	1 (0.4%)	3 (0.4%)	1881 (9.4%)
High blood pressure	22 (7.9%)	74 (8.8%)	5760 (28.7%)
Stroke	0 (0.0%)	0 (0.0%)	322 (1.6%)
Circulation problems	3 (1.1%)	5 (0.6%)	733 (3.7%)
Lung disease	1 (0.4%)	8 (1.0%)	1522 (7.6%)
Diabetes	18 (6.4%)	38 (4.5%)	1169 (5.8%)
Kidney disease	2 (0.7%)	6 (0.7%)	343 (1.7%)
Nervous system	7 (2.5%)	54 (6.4%)	218 (1.1%)
Liver disease	0 (0.0%)	0 (0.0%)	119 (0.6%)
Cancer	3 (1.1%)	11 (1.3%)	1095 (5.5%)
Depression	0 (0.0%)	0 (0.0%)	914 (4.6%)
Arthritis	0 (0.0%)	0 (0.0%)	3704 (18.5%)

^a^NHS patients whose age and sex were not included in NHS patient-level data were not included in subgroup percentage calculations.

Table 2 presents a summary overview of EQ-5D(3L) item level responses of groin hernia patients in the VCH and the NHS. Proportionally more NHS patients reported problems with pain/discomfort compared to VCH participants. Conversely, proportionally more VCH participants reported problems in the domains of mobility, self care, usual activities and anxiety/depression. These observations suggest that unadjusted preoperative health was worse among VCH groin hernia patients.

**Table 2.** Summary of EQ-5D(3L) item level responses of groin hernia patients in the VCH and the NHS.

	Preoperative			Postoperative		
	VCH	NHS (matched)	NHS (unmatched)	VCH	NHS (matched)	NHS (unmatched)
EQ-5D(3L) Item	*n* = 280 (%)	*n* = 840 (%)	*n* = 20,059 (%)	*n* = 280 (%)	*n* = 840 (%)	*n* = 20,059 (%)
Mobility						
No problems	208 (74.3%)	712 (84.8%)	16,348 (81.5%)	235 (83.9%)	730 (86.9%)	16,542 (82.5%)
Moderate problems	72 (25.7%)	126 (15.0%)	3694 (18.4%)	45 (16.1%)	109 (13.0%)	3472 (17.3%)
Severe problems	0 (0%)	2 (0.2%)	17 (0.1%)	0 (0%)	1 (0.1%)	45 (0.2%)
Self care						
No problems	259 (92.5%)	815 (97.0%)	19,334 (96.4%)	263 (93.9%)	803 (95.6%)	19,055 (95.0%)
Moderate problems	21 (7.5%)	24 (2.9%)	691 (3.4%)	16 (5.7%)	35 (4.2%)	943 (4.7%)
Severe problems	0 (0%)	1 (0.1%)	34 (0.1%)	1 (0.4%)	2 (0.2%)	61 (0.3%)
Usual activities						
No problems	173 (61.8%)	644 (76.7%)	14,698 (73.3%)	225 (80.4%)	701 (83.5%)	15,980 (79.7%)
Moderate problems	95 (33.9%)	177 (21.1%)	4932 (24.6%)	48 (17.1%)	134 (16.0%)	3829 (19.1%)
Severe problems	12 (4.3%)	19 (2.3%)	429 (2.1%)	7 (2.5%)	5 (0.6%)	250 (1.2%)
Pain/discomfort						
No problems	108 (38.6%)	292 (34.8%)	6363 (31.7%)	190 (67.9%)	571 (68.0%)	13,482 (67.2%)
Moderate problems	160 (57.1%)	514 (61.2%)	12,789 (63.8%)	82 (29.3%)	258 (30.7%)	6216 (31.0%)
Severe problems	12 (4.3%)	34 (4.0%)	907 (4.5%)	8 (2.9%)	11 (1.3%)	361 (1.8%)
Anxiety/depression						
No problems	191 (68.2%)	741 (88.2%)	17,114 (85.3%)	226 (80.7%)	753 (89.6%)	17,522 (87.4%)
Moderate problems	82 (29.3%)	97 (11.5%)	2767 (13.8%)	45 (16.1%)	83 (9.9%)	2351 (11.7%)
Severe problems	7 (2.5%)	2 (0.2%)	178 (0.9%)	9 (3.2%)	4 (0.5%)	186 (0.9%)

Postoperatively, disparities between the two systems’ groin hernia patients were smaller; the unadjusted proportion of NHS patients and VCH participants reporting problems was very similar for mobility, self care, usual activities and pain/discomfort. Nearly 9% more VCH participants reported moderate or severe problems concerning anxiety/depression compared to NHS patients.

The results of the pre- to postoperative unadjusted change in the EQ-5D(3L) VAS are shown in Table 3. Overall, VCH participants reported a statistically significantly higher mean VAS score postoperatively compared to preoperatively, indicating an improvement in overall health status. NHS patients reported a lower mean VAS score postoperatively, but this was not statistically significant.

**Table 3.** Summary statistics of preoperative and postoperative EQ-5D(3L) VAS for VCH and NHS patients.

	VCH					NHS Matched				
Characteristic	N (%)	Preoperat mean (SD)	Postoperat mean (SD)	Change mean (SD)	*p* value	*N* (%)	Preoperat mean (SD)	Postoperat mean (SD)	Change mean (SD)	*p* value
Total	280 (100%)	77.9 (15.0)	82.1 (14.9)	4.2 (15.2)	<0.01	840 (100%)	82.3 (14.3)	80.9 (16.1)	−1.41 (15.9)	0.06
Age category (years)										
20–29	2 (0.7%)	69.0 (41.0)	85.0 (14.1)	16.0 (26.9)	0.68	3 (0.4%)	81.7 (7.6)	82.7 (11.7)	1.0 (5.3)	0.91
30–39	5 (1.8%)	86.0 (10.8)	88.0 (11.0)	2.0 (5.7)	0.78	13 (1.5%)	81.4 (13.2)	80.0 (25.3)	−1.4 (30.2)	0.86
40–49	20 (7.1%)	77.6 (11.2)	82.3 (9.8)	4.7 (13.6)	0.17	53 (6.3%)	80.9 (13.1)	79.1 (16.2)	−1.8 (15.6)	0.53
50–59	51 (18.2%)	81.0 (11.1)	83.9 (11.1)	2.9 (11.0)	0.19	123 (14.6%)	80.9 (16.1)	81.3 (14.8)	0.4 (13.0)	0.82
60–69	98 (35.0%)	77.1 (14.2)	83.7 (13.7)	6.6 (15.5)	<0.01	323 (38.5%)	83.1 (13.8)	81.9 (15.1)	−1.2 (15.3)	0.29
70–79	104 (37.1%)	77.0 (17.5)	79.3 (18.0)	2.3 (17.0)	0.35	325 (38.7%)	82.3 (14.4)	80.0 (17.2)	−2.3 (16.8)	0.07
80–89	0 (0%)	–	–	–	–	0 (0.0%)	–	–	–	–
Sex										
Male	235 (83.9%)	78.1 (15.0)	82.6 (14.3)	4.6 (15.1)	<0.01	747 (88.9%)	82.3 (14.1)	80.6 (16.6)	−1.6 (15.7)	0.04
Female	45 (16.1%)	76.9 (15.0)	79.0 (17.2)	2.1 (15.7)	0.54	93 (11.1%)	82.7 (16.7)	82.9 (11.0)	0.3 (17.3)	0.89
Comorbidities										
Heart disease	1 (0.4%)	–	–	–	–	3 (0.4%)	76.7 (23.1)	75.0 (18.0)	−1.6 (16.1)	0.92
High blood pressure	22 (7.9%)	74.3 (18.4)	82.8 (12.6)	8.5 (21.2)	0.08	74 (8.8%)	82.5 (11.7)	80.1 (12.7)	−2.4 (13.2)	0.23
Stroke	0 (0%)	–	–	–	–	0 (0.0%)	–	–	–	–
Circulation problems	3 (1.1%)	67.3 (11.0)	81 (12.8)	13.7 (5.1)	0.23	5 (0.6%)	76.0 (4.2)	72.6 (20.0)	−3.4 (16.5)	0.73
Lung disease	1 (0.4%)	–	–	–	–	8 (1.0%)	81.3 (14.1)	83.4 (11.9)	2.1 (16.7)	0.75
Diabetes	18 (6.4%)	70.5 (18.1)	83.4 (15.4)	12.9 (21.5)	0.03	38 (4.5%)	79.3 (13.8)	74.2 (16.6)	−5.2 (15.1)	0.15
Kidney disease	2 (0.7%)	49.0 (43.8)	55.0 (49.5)	6.0 (5.7)	0.91	6 (0.7%)	80.0 (8.4)	79.7 (9.1)	−0.3 (5.5)	0.95
Nervous system	7 (2.5%)	62.4 (11.5)	79.9 (13.0	17.4 (11.0)	0.02	54 (6.4%)	68.2 (16.5)	62.9 (20.8)	−5.3 (17.5)	0.15
Liver disease	0 (0%)	–	–	–	–	0 (0.0%)	–	–	–	–
Cancer	3 (1.1%)	70.0 (10.0)	77.3 (14.8)	7.3 (5.5)	0.52	11 (1.3%)	85.7 (9.7)	78.1 (18.1)	−7.6 (15.1)	0.24
Depression	0 (0%)	–	–	–	–	0 (0.0%)	–	–	–	–
Arthritis	0 (0%)	–	–	–	–	0 (0.0%)	–	–	–	–

Disaggregating data at subgroup level, VCH patients who were aged 60–69 years, male, and those with either diabetes or nervous system-related comorbidities reported significantly higher mean postoperative VAS scores. This was not the case for NHS patients, where most subgroups reported a reduction in mean VAS score although this reduction was found to be statistically significant for male patients only (*p* = 0.05).

Table 4 presents the findings of the regression analysis measuring postoperative ED-5D(3L) VAS scores. This shows that VCH patients reported significantly (*p* < 0.01) greater postoperative VAS scores (better health) compared to their matched sample of NHS patients. Higher preoperative scores were associated with higher postoperative scores (*p* < 0.01). No differences were observed for age categories or sex. Only one comorbidity, nervous system disorders, was statistically significantly associated with poorer postoperative health (*p* < 0.01), although the small number of participants reporting this comorbidity indicates that more research is needed to confirm this finding.

**Table 4.** Regression analysis results measuring postoperative ED-5D(3L) VAS scores.

Regression variable	Coefficient (SE)	*p* value
Intercept	46.82 (6.65)	<0.01
Baseline score	0.46 (0.03)	<0.01
Age category (years)		
20–29	Reference group	
30–39	−3.89 (7.02)	0.59
40–49	−4.80 (6.42)	0.46
50–59	−2.59 (6.30)	0.68
60–69	−2.48 (6.25)	0.69
70–79	−4.64 (6.25)	0.46
Sex		
Male	Reference group	
Female	0.02 (1.30)	0.99
Comorbidities		
Heart disease	0.11 (7.17)	0.99
High blood pressure	0.45 (1.55)	0.77
Circulation problems	0.97 (5.34)	0.86
Lung disease	3.60 (4.71)	0.45
Diabetes	−1.65 (2.04)	0.42
Kidney disease	−2.91 (4.98)	0.56
Nervous system	−10.02 (1.92)	<0.01
Cancer	−4.23 (3.76)	0.26
Health system		
NHS	Reference group	
VCH	2.66 (0.99)	<0.01

## Discussion

This study took advantage of a natural experiment wherein PROMs for the same condition were collected in two health care systems in approximately similar time periods. Examples where multiple countries have collected the same outcome measures for the same intervention are lacking, and the availability of public PROMs data files from the NHS in England is unique. The value of this study lies in the identification and highlighting of challenges necessary to overcome for conducting valid international comparisons using PROMs. We here describe some of the main issues that arise from our work.

We used propensity scoring methods to match patients in the NHS and VCH to reduce differences in demographic and comorbidity characteristics. In the NHS data, patients’ comorbidities were self-reported while for the VCH, comorbidities were obtained from patients’ hospital discharge summaries. This difference in approach may result in under- or overreporting of comorbidities in either setting and difficulties in matching by case-mix, thereby amplifying the need for consistency through which comorbidities are reported to reduce potential bias in cross-national comparison. For example, in the NHS data, 4.6% of the sample of patients reported depression but no such cases were recorded in the discharge summaries of VCH patients considered in the analysis. The latter is likely attributable to perceived lack of relevance to the patient’s surgical episode and is important in that the matching algorithm likely resulted in fewer comorbidities reported among VCH patients, biasing the matches towards healthy patients. While these problems are likely of less importance in national analyses that use standardized coding practices, international comparative work would need to take careful consideration of potentially problematic variables whose definitions or interpretations vary between settings.

Similar issues arose for age, with the NHS dataset using categorial variables to anonymize data, and coding systems. The NHS dataset was based on OPCS-4 codes for interventions whereas the Canadian dataset used CCI codes. We sought to address this difference through mapping classification codes; however precise mapping may prove challenging where multiple countries are involved, which may result in inexact comparisons of interventions or inability to reliably match patients between samples.

In addition, both NHS and VCH data lacked indicators of a condition’s severity, risk of requiring emergency surgery or contextualizing information. For instance, patients’ socioeconomic status, language or literacy may have played an unknown role in their perioperative care. International comparisons of PROMs should carefully evaluate the constellation of factors that influence patients’ outcomes and this study provides no insight into other health and social factors than may have impacted patients’ outcomes.

The question of the sample’s generalizability equally affects the conclusions and the approach to collecting PROMs information will be important for international comparative analyses. While we were able to draw on population-based PROMs data collection in the NHS, the VCH data relied on a pragmatic sample of consecutive patients of a number of surgeons from four Vancouver hospitals. Participation bias may this be lower among the NHS patients, whereas the VCH data may reflect variable levels of encouragement of participation by individual surgeons, with VCH participants contacted by phone, followed by up to two reminders, to complete and return their PROMs. Also, the EQ-5D(3L) is sent out six months postoperatively while in the NHS, PROMs data is collected three months postoperatively. The differences in PROMs collection processes may induce participation biases of unknown direction and are another limitation of this study.

As a result, our findings have to be framed in the context of different sampling designs. Although similar in this study, the data was not collected at exactly the same time, nor with the same follow-up protocols. When the time of administration varies, there is the possibility of response shift, or changes in patients’ perceptions of their problems. In a stronger design, the survey administration times would be synchronized between settings and protocols for follow-up and non-response would be aligned and reflected in the analytic methods.

This study also highlighted the importance of the instrument for data collection for cross-country comparative purposes. In this study, the comparisons were based on the EQ-5D(3L), a generic PROM instrument. While sufficient for a general comparison of health status, a more detailed analysis of improvements in hernia-specific symptoms would require health systems to use the same condition-specific instrument. While there is recognition that using both a generic and condition-specific instrument will be needed for many health conditions,^18^ international comparisons would require consensus on the generic and condition-specific instruments to be used.

There are lessons regarding feasibility and practice to be learned from multinational randomized controlled trials that have used standardized PROMs irrespective of the setting.^17^ Other international efforts, such as the International Consortium for Health Outcomes Measurement, have supported standardized sets of instruments to facilitate cross-country comparisons. However, there is as yet no consensus on measures for hernia repair and this lack of progress reveals the difficulty in gaining support for condition-specific PROM instruments between surgeons, hospitals and health care systems more broadly.

Our study observed a statistically significant improvement in postoperative health status among VCH patients but not among NHS patients. But given the problems around generalizability and comparing performance as described above, the statistically significant difference may not even be clinically meaningful. Further research is required into postoperative recovery and rehabilitation trajectories in the two systems to attribute any observed change to surgery rather than the continuum of perioperative care or patients’ other contextual factors, such as social vulnerabilities. We further accept that predictors of outcomes may be specific to each country’s health and social care systems.^22^

In conclusion, we note that the actual findings of this study are perhaps less important than the broader lessons learned and its implications for future research. Our study does not answer how the PROM data should be interpreted for the purpose of comparing surgical performance between the two systems. Yet, despite the limitations of comparing PROMs as identified in this study, cross-country comparisons of patient-centred outcomes show considerable promise, supported by an impetus for international comparisons of health care systems’ performances incorporating PROMs. Significant methodological work remains for this kind of comparative analysis to be done on a larger scale, and this exploratory study provides pointers for a roadmap to conduct cross-country comparisons using PROMs, including instrument selection, survey administration, case-mix/risk adjustment and variable selection, coding systems, and postoperative follow-up.
